# RSV Promotes Epithelial Neuroendocrine Phenotype Differentiation through NODAL Signaling Pathway

**DOI:** 10.1155/2021/9956078

**Published:** 2021-09-08

**Authors:** Dan Liu, Zhongxiang Tang, Kezi Qiu, Ousman Bajinka, Lili Wang, Ling Qin, Yurong Tan

**Affiliations:** ^1^Department of Medical Microbiology, School of Basic Medical Sciences, Central South University, Changsha, 410078 Hunan, China; ^2^China-Africa Research Center of Infectious Diseases, School of Basic Medical Sciences, Central South University, Changsha, 410078 Hunan, China; ^3^Department of Respiratory Medicine, Xiangya Hospital, Central South University, Changsha, 410078 Hunan, China

## Abstract

**Background:**

Respiratory syncytial virus (RSV) infects infants and children, predisposing them to development of asthma during adulthood. Epithelial neuroendocrine phenotypes may be associated with development of asthma. This study hopes to ascertain if RSV infection promotes epithelial neuroendocrine phenotypes through the NODAL signaling pathway.

**Methods:**

The GSE6802 data set was obtained from the GEO database, and the differential genes were analyzed using the *R* language. An in vitro model was constructed with RSV infected human respiratory epithelial cells, and then real-time qPCR and immunofluorescence were used to detect the expression of different epithelial biomarkers and airway neuropeptides. The acute and chronic infection model of RSV infection was established by intranasal injection of RSV into guinea pigs. Immunohistochemistry and Western blot were used to detect the expression of pulmonary neuroendocrine cells markers ENO2 and neuropeptides.

**Results:**

The expression levels of ENO2, SP, CGRP, and NODAL/ACTRII were significantly higher in the RSV infection group than those of the control group, which were abrogated by siRNA-NODAL. In vivo, we found that the expression levels of ENO2, SP, and CGRP were significantly higher than that of the control group.

**Conclusion:**

RSV promotes epithelial neuroendocrine phenotypes through the NODAL signaling pathway.

## 1. Introduction

Respiratory syncytial virus (RSV) is an enveloped RNA virus with a single-stranded negative chain genome. It is a common pathogen of lower respiratory tract, infecting infants and young children and comes with symptoms such as coughing, wheezing, and breathing difficulties. Infection with RSV in infancy promoted the incidence of asthma in adulthood significantly [1]. Airway hyperresponsiveness (AHR) is one of the key pathological characteristics of asthma, which is characterized by the excessive or premature contractile response of airways to various stimulants and plays a very important role in the diagnosis and prognosis assessment of asthma [2]. The mechanism of AHR is very complicated, and it is generally considered to be related to genetic susceptibility, airway inflammation, Th1/Th2 imbalance, neuromodulator, and airway smooth muscle abnormalities and remodeling [3].

Of recent, the relationship between neuropeptide-mediated neurogenic inflammation and asthma is attracting much attention in the scientific research. Neuropeptides are mainly produced by nonadrenergic noncholinergic (NANC) sensory nerves and pulmonary neuroendocrine cells (PNECs). The bronchial epithelium is composed of ciliated cells, goblet cells, basal cells, brush cells, Clara cells, and PNECs. The PNECs contain a variety of active amines and peptides, such as 5-hydroxytryptamine, calcitonin, calcitonin gene-related peptide (CGRP), and enkephalin [4]. These secretions may be involved in regulating the contraction of airway vascular smooth muscle through paracrine action or blood circulation. Joad et al. [5] found that the number of PNECs around the bronchi was significantly related to AHR. Another study showed that the expression of substance *P* (SP) increased significantly after RSV acute infection [6] . Our previous studies also confirmed an increase of pan-neural marker PGP9.5, SP, and CGRP in the airways of guinea pigs after RSV infection and a decrease in the expression of vasoactive intestinal peptide (VIP) [7]. RSV can also cause neurological remodeling in the lungs and brain through the brain-gut axis [8]. However, the mechanism of RSV infection-induced neurogenic remodeling and the changes in airway pathophysiology has no scientific ground, or it is still in the mystery.

NODAL is an important part of the TGF-*β* family, which is mainly expressed in embryonic tissue and is generally not expressed in normal adult tissue [9]. Recent studies have shown that NODAL is expressed in a variety of malignant tumors, and that it is closely related to the occurrence, development, leaching, and metastasis of tumors as an epithelial-mesenchymal transition (EMT) regulatory gene. NODAL can activate the Smad 2/3 protein by binding type I or type II serine/threonine kinase receptors through paracrine and self-secretion, and then induce the phosphorylation of Smad 2/3 complex to bind to the Smad 4 protein and their transportation to the nucleus, and then regulate the transcription of the target genes by binding to DNA, thereby promoting cell proliferation and EMT [10, 11]. Our previous research showed that infection of bronchial epithelial cells (BECs) by different microorganisms will show effects on the expression of NODAL. For example, under the action of inactivated *P. aeruginosa* and *A. baumannii*, the expression of NODAL is inhibited, while RSV infection promotes the expression of NODAL. The upregulated NODAL may participate in the development of AHR by promoting the differentiation of Th2 and Th17 and inhibiting the differentiation of Th1 cells, thus inducing airway inflammation and AHR [12]. Furthermore, we found that RSV-infected BECs could affect the differentiation of helper T cells in the airway microenvironment, and the Th2 inflammatory response can induce AHR and accelerate the development of bronchial asthma [13].

Using bioinformatics to analyze RSV-infected BECs, we found that RSV infection can promote the expression levels of PNECs marker enolase-2 (ENO2) and NODAL/ACTRII in BECs. Going with our previous study, RSV infection promotes airway epithelial cell differentiation through NODAL/ACTRII. We opted to study whether RSV infection promotes epithelial differentiation into neuroendocrine phenotypes through the NODAL signaling pathway. This is imperative to clarify the epithelial differentiation induced by RSV infection and its related mechanisms, and it can also provide new clinical ideas for prevention and treatment of AHR.

## 2. Materials and Methods

### 2.1. Data Preprocessing and Differentially Expressed Gene Screening

The data set GSE6802 was obtained from the Gene Expression Omnibus (GEO) database (https://www.ncbi.nlm.nih.gov/geo/).The data set GSE6802 is the mRNA expression profile of BECs (BEAS-2B) treated by different microorganisms and the samples including 3 controls and 3 samples infected with RSV strain A2 (multiplicity of infection (MOI) = 1) for 4 h. *R* software (version 3.5.1; https://www.r-project.org/) and bioconductor (http://www.bioconductor.org/) was used to process raw data. After the data set was calibrated and normalized, the limma package was used for screening (∣log2FC | ≥2, *p* value ≤ 0.05).

### 2.2. Cell Culture and RSV Infection

Human BECs (BEAS-2B) and Hela were cultured in DMEM containing 10% fetal bovine serum (FBS) at 37°C, and 5% CO_2_. RSV (A2 type) was stored by the Department of Microbiology, Central South University. 70% confluent of Hela cells was infected with 100 *μ*L of RSV for 2 h at 37°C [14, 15], and then the nonabsorbed virus was removed and washed twice. Thereafter, the cells were cultured in DMEM with 10% FBS. When the cytopathic effect reaches maximum, the cells are repeatedly frozen and thawed three times, then centrifuged at 1000 rpm for 5 minutes to remove debris and filtered with a 0.22 *μ*m filter. The collected virus was tested for 50% cell culture infectious dose (TCID50) and stored at -80°C until use. RSV infection was verified by indirect immunofluorescence (IFA) with RSV major surface glycoprotein G monoclonal antibody (Bioss, bs-1264R, Beijing, China) as a primary antibody and FITC-conjucted antibody (Boster, BA1105, Wuhan, China) as secondary antibody.

### 2.3. NODAL Knockdown by siRNA and RSV Infection

The knockdown of NODAL in BECs was achieved by transfection of NODAL siRNA, and control siRNA was purchased from Ribobio (Shanghai, China). Seed cells were inoculated into 6-well plates at 10^6^ cells per well and cultured to 30-50% confluence. Then, a final concentration of 20 *μ*M siRNAs was transfected into cells for 48 h by riboFECT CP Transfection Kit (166 T), and then the medium to antibiotic-free medium BEGM was changed. Subsequently, the cells were infected with RSV at MOI of 0.2. After 2 h of RSV adsorption, the viral suspension was removed, cells washed twice, and the cells were then cultured in a fresh medium containing 2% FBS.

### 2.4. Animal Models

One-month-old female guinea pigs weighing 150–200 g (purchased from Department of Laboratory Animals, Xiangya School of Medicine, Central South University) were kept in a pathogen-free environment. Four healthy guinea pigs were randomly selected and executed, and their lungs were taken for follow-up studies. The remaining guinea pigs were then randomly divided into the control group (*n* = 12) and RSV group (*n* = 12). After anesthetizing the guinea pigs with ether, 10^6^ pfu of RSV in 100 *μ*l was intranasally inoculated. The guinea pigs were sacrificed on days 7 and 28 of postinfection with 6 guinea pigs from each group. RSV infection was assayed using IFA.

### 2.5. qRT-PCR

Primers were designed using the Primer Premier 5.0 program (Table 1) and synthesized by Dingguochangsheng Biotechnology, Beijing, China. Total RNA was extracted using TRNzol Universal Reagent (TANGEN, Beijing, China). Each sample was reverse transcribed into cDNA using *TransScript*® One-Step gDNA Removal and cDNA Synthesis SuperMix (Transgen, Beijing, China), and then the cDNA was synthesized by reverse transcription and amplified using a qPCR kit (TansGen Biotech Technology Co., Ltd., Beijing, China) according to the manufacturer's instructions. qPCR was performed at 94°C for 3 min and 35 cycles of 94°C for 45 s, 51°C for 45 s, and 72°C for 45 s. GAPDH was used for normalization, and relative quantity of the mRNA expression was calculated by using the 2^−*ΔΔ*CT^ method.

### 2.6. Hematoxylin-Eosin Staining

The left lung tissues taken from each animal were fixed in 10% formaldehyde solution for 24 h and then embedded in paraffin wax blocks. We stained 5 *μ*m paraffin sections with HE, and observed the pathological morphology under an optical microscope.

### 2.7. Immunofluorescence

The BECs were fixed by 95% ethanol and 0.1% Triton-X100. The samples were then blocked with normal goat serum for 20 min and incubated overnight with SP (Bioss, bs-0065R, Beijing, China), VIP (Bioss, bs-0077R, Beijing, China), or CGRP (Bioss, bs-0791R, Beijing, China) rabbit primary antibody at 4°C. Then, the samples were incubated with a FITC-conjugated goat anti-rabbit secondary antibody (Boster, BA1105, Wuhan, China) at room temperature for 1 h. After counterstained with DAPI for 10 min, the samples were observed with a laser scanning confocal microscopy. Images were acquired with ×40 objectives using a digital camera. The integral optical density values (IOD) of the VIP, SP, and CGRP protein were measured by Image-Pro Plus 6.0.

### 2.8. Western Blot Analysis

Lung tissue and cellular protein were extracted using RIPA buffer (EpiZyme, PC101, Shanghai, China) with 0.1% PMSF, lysed on ice for 30 min, and centrifuged at 12,000 r for 10 min. The protein was quantified using a BCA Kit, and a 30 *μ*g protein sample was obtained for 12-15% SDS-PAGE electrophoresis and transferred onto the PVDF membrane and then probed with rabbit anti-ENO2 (Bioss, bs-6273R, Beijing, China), Rabbit anti-SP (Beyotime, AF8094, Shanghai, China), Rabbit anti-VIP (Beyotime, AF8331, Shanghai, China), or Rabbit anti-CGRP (Beyotime, AF6495, Shanghai, China) primary antibodies overnight at 4×C. The secondary antibody of horseradish peroxidase (HRP)-goat anti-rabbit immunoglobulin G (IgG) (Boster, BA1055, Wuhan, China) was diluted to 1 : 3,000. The signals were detected using ECL (Millipore, WBLUR0100, Millipore, USA). The bands were visualized with the chemiluminescence detection system (Tanon 5200 Multi, Beijing, China), and the protein expression levels were normalized to GAPDH levels. The integrated density of proteins was quantified using ImageJ software (NIH).

### 2.9. Immunohistochemistry

Following routine deparaffin and hydration, sections were treated with 3% H_2_O_2_ at room temperature for 5-10 min to inactivate endogenous enzymes. After heating the 0.01 M sodium citrate buffer (pH 6.0) to about 95×C, brought the sections to a boil in it for 20 min, and the blocking solution was added at room temperature for 20 min. The sample was incubated overnight at 4×C with rabbit anti-ENO2 polyclonal antibody (Bioss, bs-6273R, Beijing, China), then incubated with goat anti-rabbit IgG antibody and streptavidin-POD working solution in turn. Subsequently, the slides were stained with DAB staining solution for 15 min, counterstained with hematoxylin, and then observed under a light microscope. Images were acquired with ×10 objectives using a digital camera. The IOD of the ENO2 protein was measured by Image-Pro Plus 6.0.

### 2.10. Statistical Analysis

SPSS21.0 software was used for statistical analysis, and the data were expressed as mean ± standard deviation. *t*-test was used for comparison between two groups, two-way ANOVA was used for comparison among multiple groups, and LSD was used for posthoc test. *p* < 0.01 was considered significant.

## 3. Results

### 3.1. Different Expression of mRNA Levels between Uninfected and RSV-Infected BEAS-2B Cells

**Figure 1:**
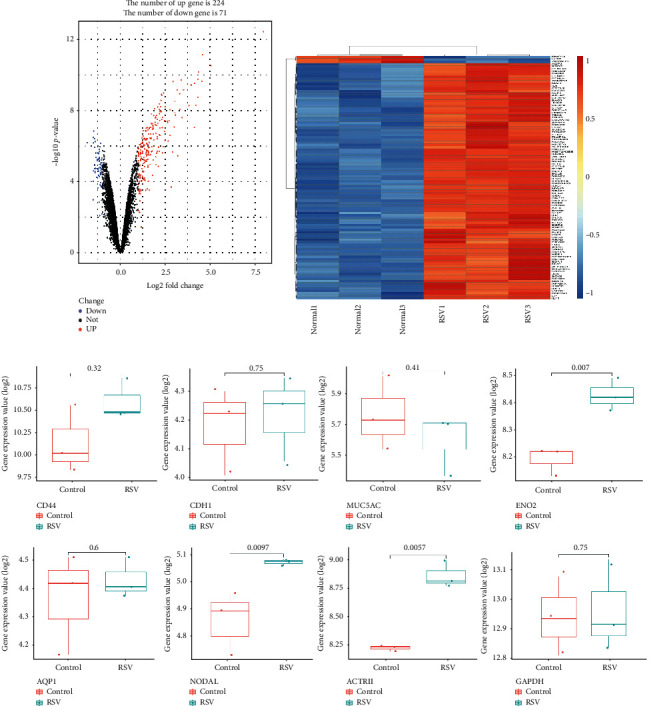
Different expression of mRNA levels between uninfected and RSV-infected BEAS-2B cells from the data set GSE6802. (a) Volcano map for 295 different expression mRNAs. Dots in red and blue indicate high and low expression of mRNA in RSV-infected BEAS-2B cells. (b) Heat map of mRNA expression profile in uninfected and RSV-infected BEAS-2B cells. (c) Expression levels of different genes in uninfected and RSV-infected BEAS-2B cells (*n* = 3). The value was expressed as log2 fold change.

From the data set GSE6802, we screened 295 significantly different genes, of which 224 were upregulated and 71 were downregulated (Figure 1(a)), and the heat map showed that these genes showed significantly different distributions in uninfected and RSV-infected BEAS-2B cells (Figure 1(b)). Then, we focused on the analysis of different epithelial cell markers, and the results showed that the expression of PNECs markers (ENO2) increased after RSV infection, while there were no significant difference in basal cell markers CD44, ciliated cells markers cadherin 1 (CDH1), goblet cell markers mucin 5 AC (MUC5A), and alveolar epithelial type I (AECI) cell markers aquaporin 1 (AQP1) (Figure 1(c)). Moreover, NODAL and its receptor ACTRII were also significantly increased (Figure 1(c)).

### 3.2. RSV Promotes Epithelial Cells to Differentiate into Neuroendocrine Phenotypes through the NODAL Signaling Pathway

**Figure 2:**
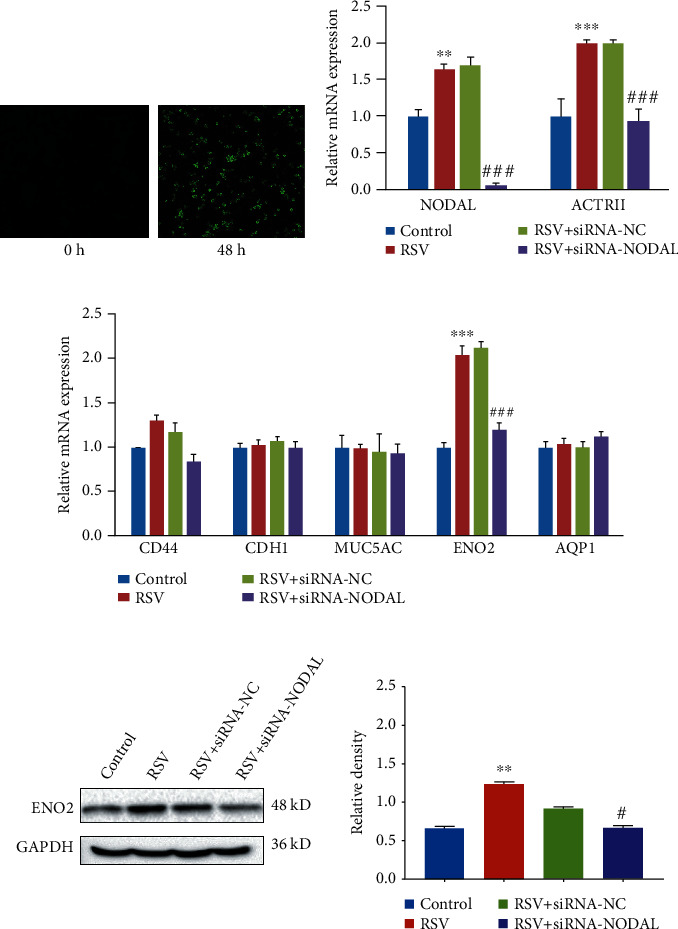
RSV promotes epithelial cells to differentiate into neuroendocrine phenotypes through the NODAL signaling pathway. (a) RSV infection in BECs was verified by indirect IFA with RSV F protein monoclonal antibody (magnification ×100). (b) The expression levels of NODAL and ACTRII in BECs were assayed using qRT-PCR (*n* = 5). (c) The mRNA levels of epithelial markers including PNECs marker ENO2, basal cell marker CD44, ciliated cells marker CDH1, goblet cell marker MUC5A, and alveolar epithelial type I ell marker AQP1were assayed using qRT-PCR (*n* = 5). (d) Western blot detection of the protein level of ENO2 in BECs. *t*-test was used for comparison between two groups (^∗∗^*p* < 0.01 and ^∗∗∗^*p* < 0.001 vs. control; #*p* < 0.05 and ###*p* < 0.001 vs. RSV+ siRNA-NC).

In order to detect the expression of NODAL signaling pathway, we infected BECs with RSV for 48 h and confirmed the infection using IFA. Compared with uninfected cells, green fluorescence was seen in the BECs (Figure 2(a)). Then, we tested the mRNA levels of NODAL and its receptors using qRT-PCR. The results showed that the expression levels of NODAL and its receptor ACTRII were significantly higher in the RSV infection group than those in the control group. Moreover, after interfering with siRNA-NODAL prior to RSV infection for 48 h, the mRNA expression levels of NODAL and ACTRII were significantly reduced compared to the siRNA-NC group (Figure 2(b)).

Next, we investigated whether the differentiation of PNECs induced by RSV infection was related to NODAL signaling molecules. The results of qRT-PCR showed that when compared with the siRNA-NC group, the expression levels of ENO2 mRNA in the siRNA-NODAL group were significantly reduced (Figure 2(c)). The results of Western blot showed that RSV infection can promote the expression of ENO2 through the NODAL signaling pathway, suggesting that RSV infection may promote the differentiation of PNECs through the NODAL signaling pathway.

### 3.3. RSV Affects the Neuropeptide Expression in BECs through the NODAL Signaling Pathway

**Figure 3:**
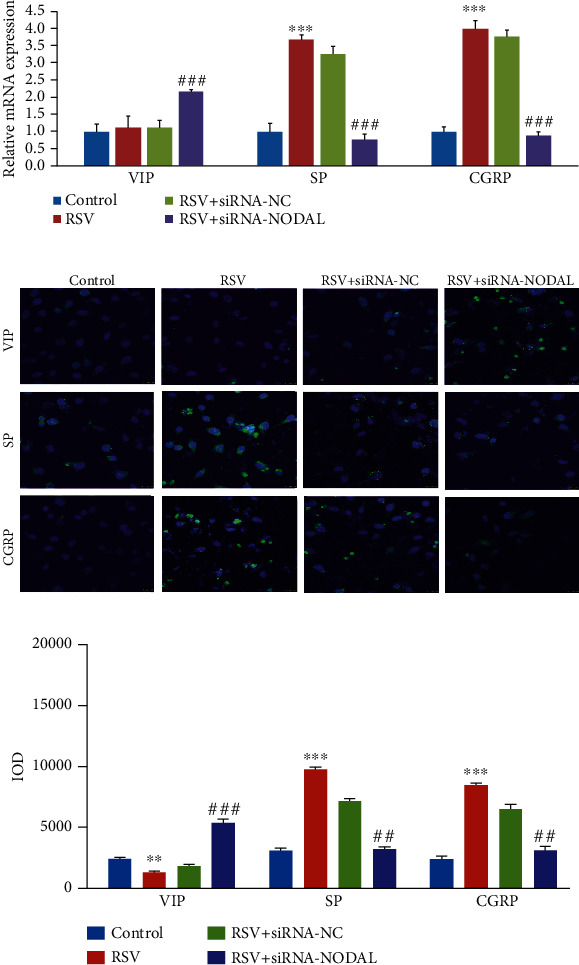
RSV affects neuropeptide synthesis in BECs cells through the NODAL signaling pathway. (a) qRT-PCR was used to detect the mRNA levels of SP, CGRP, and VIP in BECs after RSV infection (*n* = 5). After interfering with the expression of NODAL prior to 48 h of RSV infection, the expression of these three neuropeptides was detected again. (b) Immunofluorescence was used to detect the protein levels of neuropeptide in BECs (magnification ×400). *t*-test was used for comparison between two groups (^∗∗^*p* < 0.01 and ^∗∗∗^*p* < 0.001 vs. control; ##*p* < 0.01 and ###*p* < 0.001 vs. RSV+ siRNA-NC).

Then, we examined the neuropeptide expression in cells after RSV-infected BECs. The results of qRT-PCR showed that the mRNA expression of SP and CGRP increased. However, when we interfered BECs with siRNA-NODAL, prior to RSV infection for 48 h, the results showed that the mRNA of SP and CGRP was reduced, while the mRNA level of VIP increased after NODAL knockdown. This indicates that RSV can change the expression levels of intracellular neuropeptides through the NODAL signaling pathway and promote the release of excitatory neurotransmitters SP and CGRP (Figure 3(a)). In addition, we also detected the protein levels of SP, CGRP, and VIP using IFA. The results indicate that RSV infection may promote the synthesis of SP and CGRP through the NODAL signaling pathway (Figure 3(b)).

### 3.4. RSV Infection Promotes the Expression of PNEC Marker ENO2 in Lung Tissue of Guinea Pigs

**Figure 4:**
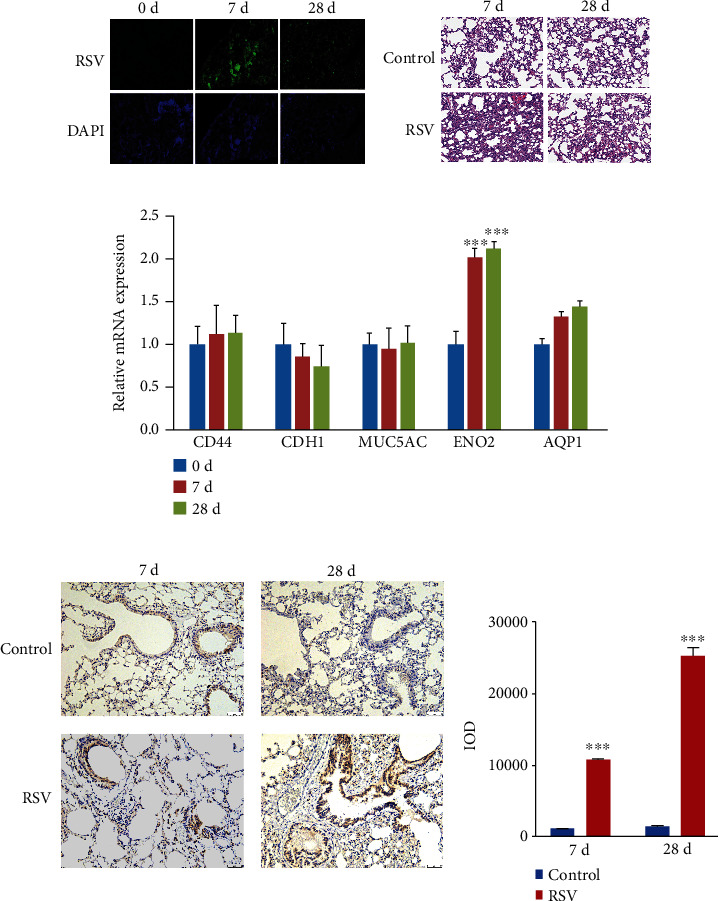
RSV infection promotes the expression of PNECs marker ENO2 in lung tissue of guinea pigs. (a), RSV infection in lung tissue of guinea pigs was assayed using IFA (magnification ×200). (b) Histology was assayed using HE staining (magnification ×100). (c) Detection of mRNA levels of different epithelial cell markers in lung tissues using qRT-PCR (*n* = 6). Two-way ANOVA was used for comparison among multiple groups, and LSD was used for posthoc test. (d) Detection of ENO2 protein level in the lung tissue of guinea pigs using immunohistochemistry. *t*-test was used for comparison between two groups (magnification ×100) (^∗∗∗^*p* < 0.001 vs. control).

The effect of RSV infection in the lungs of guinea pigs was detected with IFA, and the RSV group showed significant green fluorescence compared to the control group (Figure 4(a)). Different degrees of inflammation appeared in the lungs of RSV-infected guinea pigs. During the RSV acute infection period (7 d), the guinea pigs' pulmonary interstitial edema and epithelial cells shed and died. Microvessels and airways were infiltrated by a large number of inflammatory cells. Over time, the degree of inflammation gradually weakened during the chronic infection period (28 d) (Figure 4(b)). The expression of different epithelial cell markers in the lung tissue of guinea pigs was detected with qRT-PCR, and the results showed an increase in the ENO2 expression in the RSV infection group when compared to the control group (Figure 4(c)). Then, the protein level of ENO2 was detected using immunohistochemistry. The results showed that the expression level of ENO2 in the RSV group was significantly higher than the control group on the day 7 or 28 postinfection (Figure 4(d)).

### 3.5. Effects of RSV Infection on the Neuropeptide Expression in Lung Tissues of Guinea Pigs

**Figure 5:**
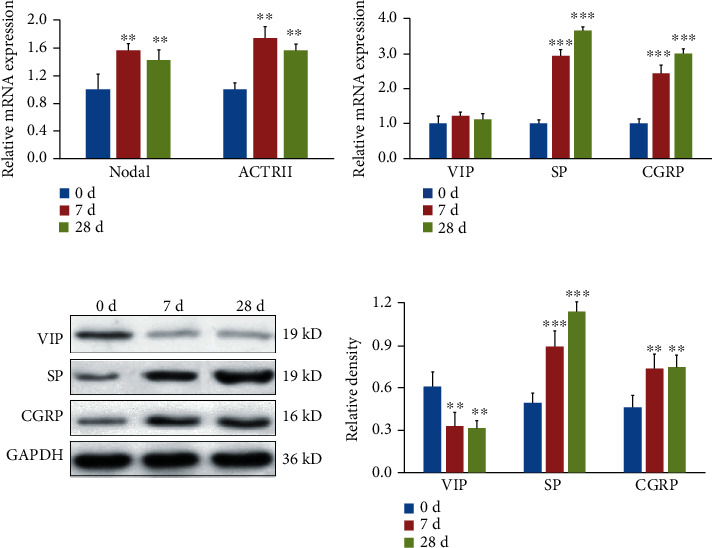
Effects of RSV infection on the neuropeptide expression in the lung tissue of guinea pigs (*n* = 6). (a) The expression levels of NODAL/ACTRII and neuropeptides in the lung tissue of guinea pigs were detected using qPCR. (b) The protein levels of neuropeptides in the lung tissue of guinea pigs were assayed using Western blot. Two-way ANOVA was used for comparison among multiple groups, and LSD was used for posthoc test. (^∗∗^*p* < 0.01 and ^∗∗∗^*p* < 0.001 vs. control).

In RSV-infected guinea pigs, the expression levels of NODAL and ACTRII in the lung tissue were upregulated (Figure 5(a)). The mRNA expression levels of SP and CGRP also increased at day 7 and day 28, while the mRNA expression level of VIP showed no difference (Figure 5(a)). The protein levels of SP, CGRP, and VIP in the lung tissue of guinea pigs were also detected using Western blot. From Figure 5(b), it can be seen that the protein synthesis of SP and CGRP in the lung of RSV-infected guinea pigs increased with the time of infection and was higher than that of the control group; however, the protein synthesis of VIP decreased with time. The above results suggested that RSV infection may promote the changes of the neuropeptide expression in the guinea pig lung tissue through the NODAL/ACTRII signaling pathway.

## 4. Discussion

Asthma is a disease that confers a public health threat over years. It is accompanied by recurrent wheezing, coughing, chest tightness, shortness of breath and other symptoms, even reversible expiratory airflow limitation [16]. Of recent, the study regarding the pathogenesis of asthma prevention and treatment measures attracts much attention in the scientific community. Asthma is characterized by chronic airway inflammation, AHR, and airway remodeling. The pathogenesis of asthma is extremely complex and has not yet been fully elucidated. Most forms of asthma are speculated to be associated with polygene inheritance and environment, including inhalants (pollen, dust, animal hair, etc.), infections (viruses, bacteria, parasites, etc.), food, and some drugs. Recent studies have found that the incidence of asthma mainly increases after viral infection including rhinovirus and RSV [17].

RSV infection can cause airway mucosal damage, increased mucus secretion, decreased ciliated cells inflammatory cell infiltration, andchanges in the ability of epithelial cells to differentiate and even affect the brain function [18]. With this study on the differentiation of respiratory epithelial cells caused by RSV infection, we found that the expression of epithelial cell marker ENO2 increased significantly after RSV infection. This suggests that RSV infection can promote the epithelial cells to differentiate into neuroendocrine phenotypes. In addition to adrenergic and cholinergic nerve innervation, bronchial asthma is also regulated by the NANC nervous system. The neurotransmitters and neuropeptides released by NANC nerves and PNECs are important factors that cause asthma. SP is a common neuropeptide, and studies have found that SP content increases in the lung tissue and bronchial lavage fluid of asthmatic guinea pigs [19]. Its biological activity mainly strongly induces airway smooth muscle contraction, promotes the secretion of inflammatory mediators and mucus, and increases AHR. CGRP is an important bioactive peptide secreted by neuroendocrine cells, and its main function is to cause airway vasodilation. Kandace [20] found that CCL17 can induce asthma and allergic diseases by releasing CGRP through CCR4 in respiratory epithelial cells. Another study found that PNECs can stimulate group II innate lymphoid cells (ILC2) to produce cytokines by producing CGRP, and these cytokines then recruit downstream immune cells. At the same time, PNECs stimulate the production of airway epithelial mucus by secreting another product, *γ*-aminobutyric acid, thereby exacerbating allergic asthma [21]. As an inhibitory nonadrenergic and noncholinergic neurotransmitter, VIP has the effects of relaxing airway smooth muscles, reducing inflammation, and regulating airway secretion in the respiratory system. Using a VIP-knockout mice model, Szema et al. [22] found that VIP is an important part of the endogenous antiasthma mechanism, and deficiency of the VIP gene may predispose to asthma. So, VIP may become an effective target for asthma. In the present study, we found that the expression levels of excitatory neuropeptides SP and CGRP increased significantly compared to those in the control group. This indicates that RSV infection promotes the increase of epithelial neuroendocrine phenotype differentiation and also leads to the change of the intracellular neuropeptide expression.

At present, the molecular biological mechanism of RSV infection affecting epithelial population differentiation and resulting in pathophysiological changes of airways is still unclear. To this effect, we analyzed RSV-infected BEC samples through bioinformatics and found that RSV infection can promote the expression of PNECs markers ENO2, NODAL, and its ACTRII receptor. Further studies verified that the expression levels of ENO2 and NODAL/ACTRII were significantly higher in the RSV infection group than that of the control group, which was abrogated by siRNA-NODAL. Moreover, the neuropeptide SP and CGRP increased in the RSV group and downregulated after siRNA-NODAL treatment. This further illustrates that RSV infection may promote BECs to differentiate into neuroendocrine phenotypes and subsequent neural network remodeling through the NODAL/ACTRII signaling pathway.

Next, the acute (7 d) and chronic (28 d) infection model of RSV infection was established by intranasal injection of RSV into guinea pigs [8, 23]. We found that during the acute infection period (7 d), epithelial cells shed in the lung tissue, and a large number of inflammatory cells infiltrated in the microvessels. The expression of ENO2 in BECs after RSV infection was significantly higher than in the control group. In addition, we found that the expression levels of SP and CGRP were significantly higher than that of the control group. The mRNA expression of VIP did not change, but the protein level decreased. We speculate that the degradation of VIP might be accelerated after RSV infection. These results showed that RSV infection induced neuroendocrine phenotypes in vivo and then might participate in the development of asthma.

In future experiments, we will continue to study the mechanism by which RSV infection may promote neural network remodeling through the NODAL/ACTRII signaling pathway. In vitro, the constructed NODAL overexpression vector will be used to study further, and the mechanism of RSV induced neuronal differentiation. In vivo, we will construct the NODAL gene knockout animal model to detect the expression changes of PNECs markers ENO2 and neuropeptide SP, CGRP, and VIP in the lungs of RSV-infected animals.

## 5. Conclusion

In conclusion, the above studies suggested that RSV can promote the differentiation of BECs into neuroendocrine phenotype through NODAL signaling pathway and thus may contribute in the development of asthma.

## Figures and Tables

**Table 1:** Primers for quantitative polymerase chain reaction.

Gene	Sequence	Length (bp)
SCGB1A1	ACTCGCTGTCACCCTCAC	18
	CCTGCCTCCCTCATGTCT	18

CD44	TGAGCATCGGATTTGAGA	18
	ATACTGGGAGGTGTTGGA	18

CDH1	CTGAGAACGAGGCTAACG	18
	GTCCACCATCATCATTCAATAT	22

MUC5AC	CCTGCCTGAAGAGCGTGAC	19
	TCTGGGCGATGATGAAGAA	19

ENO2	AGGACAAATACGGCAAGG	18
	AACTCTGAGGCAGCAACAT	19

AQP1	GCGTGACTGGAGGACTGA	18
	GAGATGGTTTGGGTGGTG	18

SPC25	CTCAATGAAGCAAGGGAC	18
	ATAAACCGTGGCAGTAAA	18

NODAL	AGTTTCATCCGACCAACCA	19
	AGGCACCCACATTCTTCC	18

ACTRII	AGACGGGAGCAGGAAAGTAAA	21
	GTAAAAGCGGTCCTAAGGAGTC	22

SP	TACCGACGTTATTATTCG	18
	CTTTATCAAAGCCTCCAT	18

VIP	AGACCCTGTACCAGTCAA	18
	CTGCTCCTCTTTCCATTC	18

CGRP	AGAATCATTGCCCAGAAGAG	20
	GGCTTTGGAACCCACATT	18

## Data Availability

The datasets used and/or analyzed during the current study are available from the corresponding author on reasonable request.
